# Dysphagia in multiple sclerosis: pathophysiology, assessment, and management—an overview

**DOI:** 10.3389/fneur.2024.1514644

**Published:** 2024-12-13

**Authors:** Domenico A. Restivo, Angelo Quartarone, Antongiulio Bruschetta, Angelo Alito, Demetrio Milardi, Rosario Marchese-Ragona, Ennio Iezzi, Sheila Peter, Diego Centonze, Mario Stampanoni Bassi

**Affiliations:** ^1^Department of Clinical and Experimental Medicine, Physical Medicine and Rehabilitation Unit, University of Messina, Messina, Italy; ^2^IRCCS Centro Neurolesi Bonino-Pulejo, Messina, Italy; ^3^Orthopedic Institute of Southern Italy “Franco Scalambrino”, Messina, Italy; ^4^Department of Biomedical and Dental Sciences and Morphofunctional Images, University of Messina, Messina, Italy; ^5^Brain Mapping Lab, Department of Biomedical and Dental Sciences and Morphofunctional Imaging, University of Messina, Messina, Italy; ^6^ENT Department, University of Padua, Padova, Italy; ^7^Unit of Neurology, IRCCS Neuromed, Pozzilli, Italy; ^8^Department of Systems Medicine, University of Rome Tor Vergata, Rome, Italy

**Keywords:** multiple sclerosis, dysphagia, swallowing rehabilitation, non-invasive brain stimulation, pharyngeal electrical stimulation

## Abstract

Dysphagia is a frequent and life-threatening complication of multiple sclerosis (MS). Swallowing disturbances may be present at all stages of MS, although their prevalence increases with age, with disease duration, and in progressive phenotypes. The pathophysiology of dysphagia in MS is likely due to a combination of factors, including the involvement of corticobulbar tracts, the cerebellum, and the brainstem. Accurate diagnosis and early management of swallowing disorders improve quality of life and may delay complications or invasive therapeutic interventions. Here we provide an overview of the pathophysiology, the assessment, and the management of MS dysphagia, also examining the possible role of novel therapeutic strategies. Although studies using imaging and neurophysiological techniques have contributed to better characterize swallowing alterations in MS, the treatment of dysphagia is still challenging. Rehabilitation represents the main therapeutic approach for swallowing disorders. Recently, some innovative neurophysiological approaches, such as pharyngeal electrical stimulation (PES), repetitive transcranial magnetic stimulation (rTMS), and transcranial direct current stimulation (tDCS), have been proposed as a supplement to swallowing therapy in different neurological conditions. However, only few studies have explored the role of neuromodulation for MS dysphagia.

## 1 Introduction

Dysphagia, the disruption of normal swallowing, is a frequent and very severe complication of multiple sclerosis (MS) (1, 2). Several clinical observations have shown that swallowing disorders are much more common in patients with MS than previously thought (2–9). Furthermore, the dysphagia-associated aspiration pneumoniae is the leading cause of death in patients with MS (9, 10).

Although dysphagia may be present at all stages of MS, the prevalence increases with age, with disease duration, and is greater in patients with progressive phenotypes (7, 8). The real prevalence of dysphagia in MS may be attested around 30–40% (7, 11). It has been reported that symptomatic dysphagia is more frequent in patients with higher Expanded Disability Status Scale (EDSS) scores and is significantly associated with cerebellar and brainstem dysfunction, as well as cognitive deficits (4, 6, 8, 12). However, about 17% of patients with low EDSS had dysphagia (2, 6). Prevalence of dysphagia increases using instrumental evaluation (8, 13). In a recent study using Fiber Endoscopic Evaluation of Swallowing (FEES), dysphagia has been detected in about 60% of MS patients (8).

Accurate diagnosis and assessment of swallowing severity and early management of dysphagia complications improve quality of life and critically contribute to delaying life-threatening complications or invasive therapeutic interventions.

## 2 Dysphagia in MS

### 2.1 Anatomy and physiology of swallowing

Swallowing is a physiological innate mechanism allowing the ingestion of liquids and foods without aspiration (14). It is commonly divided into three phases: oral, pharyngeal, and esophageal.

Swallowing is initiated by the cerebral cortex and regulated by a central pattern generator (CPG) located bilaterally in the medulla oblongata, involving several brainstem motor nuclei (V, VII, IX, X, and XII) and two major groups of interneurons: one located in the dorsal medulla within the nucleus tractus solitarius (NTS) and adjacent reticular formation, and the other one, placed in the ventrolateral medulla, just above the nucleus ambiguous (14).

Swallowing activates a large bilateral cortical–subcortical network, involving sensorimotor regions, and areas associated with cognitive, attentional, and emotional aspects (15–20). Projections from sensorimotor areas are directed toward ipsilateral and contralateral CPGs of swallowing (17). In addition, the role of the cerebellum has been consistently evidenced by studies using PET, fMRI, and transcranial magnetic stimulation TMS (21) and is confirmed by the occurrence of dysphagia in patients with cerebellar dysfunction (22, 23).

### 2.2 Pathophysiology of dysphagia in MS

The pathophysiology of dysphagia in MS is likely due to the combination of several factors such as involvement of the corticobulbar tract, the cerebellum and the brainstem, leading to impairment of one or more of the three phases of swallowing (12, 24, 25).

In patients with MS, inflammatory lesions located at the level of the cerebral hemispheres or brainstem can alter the normal physiology of swallowing, leaving the airway vulnerable to food fluid aspiration (3). Demyelinating lesions can alter swallowing when the dominant side of brain areas relevant to swallowing is affected, or when both sides of brainstem swallowing centers are affected (3). The likelihood of swallowing-relevant brain areas/connections being involved rises with increasing lesion load and the number of brainstem nuclei affected. In MS patients with mild disability but suffering from dysphagia, lesions are most likely located in regions strategically relevant to swallowing, such as medullary CPGs (2).

The cerebellum may represent another key region involved in swallowing dysfunction in MS. Notably, the cerebellum controls the output for motor nuclei of V, VII and XII cranial nerves and is an effective coordinator of muscle activation during swallowing (14, 26–28). Videofluoscopic studies in dysphagic MS subjects with cerebellar involvement have showed that cerebellar lesions are associated to dysfunction in oral phase and pharyngeal phase of swallowing (29). Other factors that might play a role in MS-related dysphagia include weakness and changes in the stiffness and elasticity of craniofacial muscles involved in swallowing, often associated to demyelination of cranial nerves, lesions in the cerebellar peduncle, internal capsule, or the spinal cord (30).

## 3 Assessment of dysphagia

### 3.1 Clinical examination

A general physical examination should be focused on the cranial nerves that are associated with swallowing, particularly the motor components of V, VII, IX, X and XII cranial nerves, and sensory fibres from V, VII, IX, and X cranial nerves (25, 31). The aspiration in patients with dysphagia may be predicted by the bedside swallowing evaluation (BsSE) (2). Water swallowing tests, such as the timed water swallowing test (TWST), are commonly used to identify patients at risk of dysphagia in different neurological conditions (32, 33), and have been evaluated as screening tools in MS (1, 2, 34).

The identification of a simple, rapid, and easy-to-implement instrument that can be used in the outpatient setting should be the ideal goal for dysphagia assessment. We recently developed a questionnaire for the evaluation of dysphagia in MS (Dysphagia in Multiple Sclerosis – DYMUS). The DYMUS is a self-administered questionnaire designed to assess signs and symptoms of dysphagia for both solids and liquids. It consists of 10 items requiring a “yes” or “no” response and can be completed in a few minutes. DYMUS scores were significantly higher in progressive phenotypes and significantly correlated with EDSS (6). Thus, the DYMUS can be used for the preliminary selection of patients for further specific instrumental analyses, and for referring patients to aspiration prevention programs (6, 8). Notably, a study evidenced that combining the TWST and the DYMUS was associated with increased sensitivity and specificity for identifying MS patients with dysphagia (34).

### 3.2 Instrumental examination

Investigation of dysphagia may include endoscopic evaluation using FEES, videofluoroscopy (VFS) and electromyography (EMG). VFS and FEES are the most used instrumental tests to identify dysphagia and represents the first-line instrumental investigation (*gold-standard*) to assess the presence of swallowing problems in MS patients (35, 36). VFS allows a dynamic evaluation of the entire swallowing act and is useful to identify the presence, nature, and severity of oropharyngeal swallowing problems, and to precisely assess the level of penetration and/or aspiration (35, 36). In the FEES, a flexible endoscope is advanced through the nose to evaluate the competence of the velo-pharyngeal sphincter and the morphology, motility and reflexes of the hypopharyngeal-laryngeal area (8, 35). FEES allows the identification of swallowing abnormalities and laryngeal penetration or endotracheal aspiration. It has been evidenced that in MS patients, FEES can identify subclinical swallowing impairment (7, 8).

EMG of the oral floor and/or pharyngo-esophageal muscles has also been employed to study swallowing disorders in different neurological diseases (12, 17, 37–39). The EMG activity of the suprahyoid/submental muscles (a muscle complex consisting of the mylohyoid, the genioglossus, and the ventral belly of the digastric muscle) marks the beginning of the propulsive action of the tongue in the oral phase of swallowing and continues throughout the pharyngeal phase (12). The EMG activity of the cricopharyngeal (CP) muscle (a structure that functionally is associated to UES) can be assessed by a needle electrode directly inserted into the muscle (37–39). At rest, a tonic activity related to its function as a muscular sphincter is recorded. Such EMG activity completely disappears (inhibitory period), for a brief time (about 0.6–1 s) during the pharyngeal phase of swallowing, leaving the bolus transit into the upper esophageal tract (13). It has been shown that electrophysiological alterations of the oro-pharyngeal phase can be frequently detected in MS patients without dysphagia and are more pronounced in progressive phenotypes (12). Prolongation of the oro-pharyngeal phase, related to incoordination in the propulsive activity was the most frequent finding (12). In addition, reduced or absent relaxation of the CP muscle of the UES has been observed in a significant proportion of MS patients (12). Reduced inhibition of the CP muscle in MS may be related to the disruption/dysfunction of swallowing circuits located in the brainstem (CPG), with consequent impairment in the propulsive activity of pharyngeal muscles (12).

### 3.3 Quantification of dysphagia severity and outcome assessment

To quantify dysphagia severity, the results of the videofluoroscopic and/or FEES examinations can be scored by the Penetration Aspiration Scale (PAS) (40). This is an eight-point scale (1 = material does not enter the airway; 8 = material enters the airway, passes below the vocal folds, and no effort is made to eject) which is largely used for semi-quantitatively assessing the degree of endoscopically and radiologically measured penetration/aspiration (40). Dysphagia outcome severity scale (DOSS) is another tool to quantify the results obtained from FEES for detecting Swallowing Deficits (7, 8). DOSS is a 7 point scale range from level 1 (Severe dysphagia) to level 7 (normal swallowing) (7, 8). Penetration and aspiration are usually chosen for measurement in clinical practice because they are associated with a more severe swallowing impairment than other traditional signs of dysphagia and because changes in penetration and aspiration are usually considered as treatment goals. Both the PAS and DOSS have been used to assess the severity of dysphagia in patients with MS (8, 41). Other important endpoints are nutritional measures such as the body mass index (BMI), and the weight loss/gain. To assess the activity limitation and participation restriction due to dysphagia, the dietary levels/restrictions and cueing may be used. The dysphagia-related quality of life (QoL) in patients with MS can be evaluated by the SWAL-QOL and SWAL-CARE (42).

## 4 Treatment

### 4.1 Rehabilitation therapy

The management of swallowing disorders in MS should be focused on the specific dysphagic symptom and the underlying pathophysiology. The goals of treating dysphagia are to improve the ability to eat and swallow, reduce aspiration, and optimise nutritional status.

Swallow therapy can be divided into compensatory techniques (i.e., postural manoeuvres), indirect therapy (exercises to strengthen swallowing muscles or by stimulating the faucial arches) (43) and direct therapy (exercises to be performed during swallowing). In addition, functional swallowing therapies have been used in neurogenic dysphagia of different aetiologies (44). For example, stimulation of the anterior faucial pillar has been used to treat dysphagia in patients with stroke (45). It has been suggested that combining mechanical, thermal, and gustatory stimulation may be more effective than thermal stimulation alone; however, the role in MS dysphagia remains unproven (44). The development of an evidence-based treatment, which can be performed repeatedly and noninvasively to reduce aspiration, is therefore crucially important. When swallowing impairment becomes more severe, enteral nutrition should be considered.

### 4.2 Pharmacological treatment

No specific pharmacological treatment for MS-associated dysphagia has been reported to date. Dysphagic MS patients may present with hypo- or hypersalivation. Both symptoms may contribute to worsen dysphagia and may benefit from artificial saliva and/or substances promoting salivation (e.g., citric acid) or by reducing saliva production with botulinum toxin type A (BoNT/A) injection into the major salivary glands (46).

If swallowing problems do not resolve within 6 months of rehabilitative or pharmacological therapy, in neurologic patients with oropharyngeal dysphagia due to UES hyper-activity, surgical myotomy of the CP muscle of UES should be considered (47). In the last year, chemical myotomy of the UES by using BoNT/A injection into the hyperactive CP muscle has been proposed as a less invasive alternative to surgical myotomy (34, 48). Chemical denervation by BoNT/A has shown to be effective in treating oropharyngeal dysphagia associated to UES hyperactivity due to different neurological disorders. We reported a beneficial effect of BoNT/A in oropharyngeal dysphagia associated with MS (49). BoNT/A injected percutaneously into the hyperactive cricopharyngeal muscle of 14 dysphagic MS patients under EMG monitoring was associated with significant improvement in swallowing outcome measures (49). These preliminary findings suggest a potential benefit from BoNT/A treatment in MS patients with dysphagia associated with UES hyperactivity.

### 4.3 Pharyngeal electrical stimulation

Recently, peripheral pharyngeal electrical stimulation (PES) in humans has provided some insight into the possible mechanisms of functional swallowing therapies and represents a useful technique for treating dysphagia of different aetiologies (50–52).

**Figure 1:**
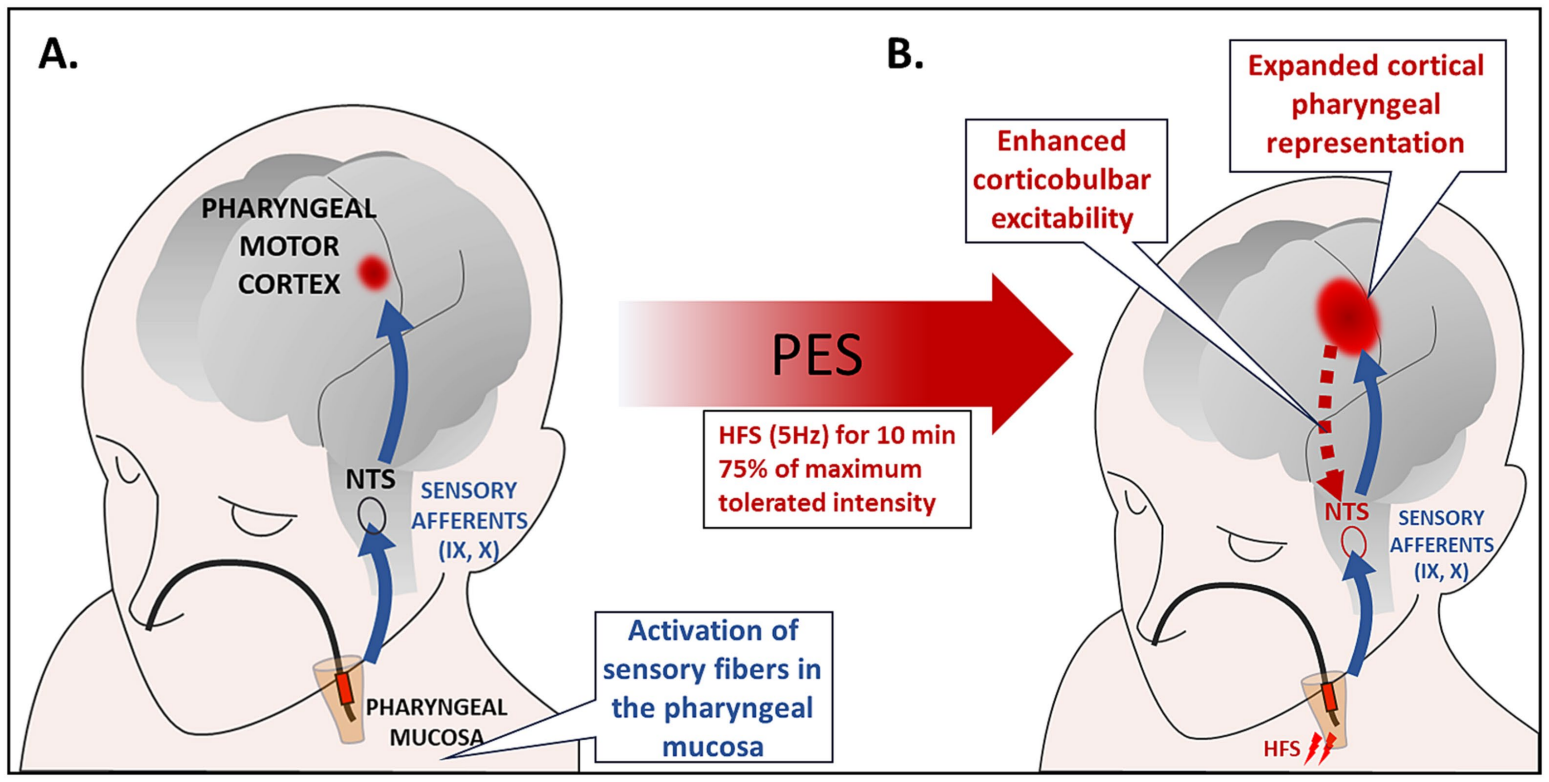
**(A)** Intraluminal electrical stimulation of the pharyngeal mucosa applied by using bipolar ring electrodes inserted through the nasal passage to the pharynx. PES activates sensory fibers in the oropharyngeal mucosa. The sensory afferents of the IX and pharyngeal branch of the X cranial nerve are directly connected to the NTS, and they also send sensory signals to cortical and subcortical areas. Neurophysiological studies evidenced that 10 min of 5 Hz stimulation induced the largest increase in corticobulbar excitability. **(B)** Studies in healthy subjects and patients with neurogenic dysphagia have demonstrated a sustained increase in cortico-bulbar excitability, remodeling of pharyngeal cortical representations, and improved swallowing function following PES. These effects have been interpreted as depending on synaptic plasticity mechanisms triggered by the convergent activity of different pharyngeal afferent inputs resulting in increased excitability of the sensorimotor swallowing cortex. HFS, high frequency stimulation; NTS, nucleus tractus solitarius; PES, pharyngeal electric stimulation.

It has been shown that high-frequency intraluminal PES (Figure 1), at an intensity just above the perception threshold, can induce a prolonged increase of pharyngeal representation in the motor cortex of healthy humans. In addition, pharyngeal electrical stimulation, applied for 10 min at 5 Hz and 75% of maximum tolerated intensity to acutely dysphagic stroke patients expanded cortical maps of pharyngeal representation, enhanced excitability of pharyngeal corticobulbar projections, improved swallowing function and reduced frequency of aspiration for at least 1 h after PES (50, 51).

The effects of pharyngeal stimulation are likely due to the activation of sensory fibers in the naso- and oropharyngeal mucosa (50). These fibers represent the sensory afferents of IXth and pharyngeal branch of Xth cranial nerves that are directly connected to the NTS (14). In addition, sensory signals are transmitted to other upper brainstem structures as well as to subcortical and cortical areas. Notably, the convergent activity of different pharyngeal afferent inputs can enhance swallowing sensorimotor cortex excitability (52). Changes in the excitability of the swallowing cortex following PES, and the resulting rearrangement of neural organization, are considered mechanisms of cortical plasticity (52, 53).

Restivo et al. (53) investigated the effect of PES on swallowing recovery in 20 MS patients with severe dysphagia. Patients were randomized to receive 5 Hz “real” PES or “sham” stimulation for 10 min for 5 consecutive days (53). Patients who received “real” PES showed a significant reduction of the PAS score that was the primary study outcome as well as a significant improvement in all the swallowing secondary outcome measures analyzed as compared with those receiving “sham” stimulation (53). These preliminary results have been interpreted as short-term stimulus-induced cortical plasticity, resembling the long-term changes observed in post-stroke patients, and the study suggests a potential benefit of PES for MS-associated dysphagia. Further studies are needed to evaluate the effects of PES in MS patients with different swallowing impairments, and particularly in dysphagia associated with UES hyperactivity. Another important aspect is the side of stimulation catheter position and consequently the site of stimulation (54).

### 4.4 Non-invasive brain stimulation techniques

Non-invasive brain stimulation (NIBS) represents a promising approach to improve motor and sensory deficits and boost the effects of neurorehabilitation in MS (55).

**Figure 2:**
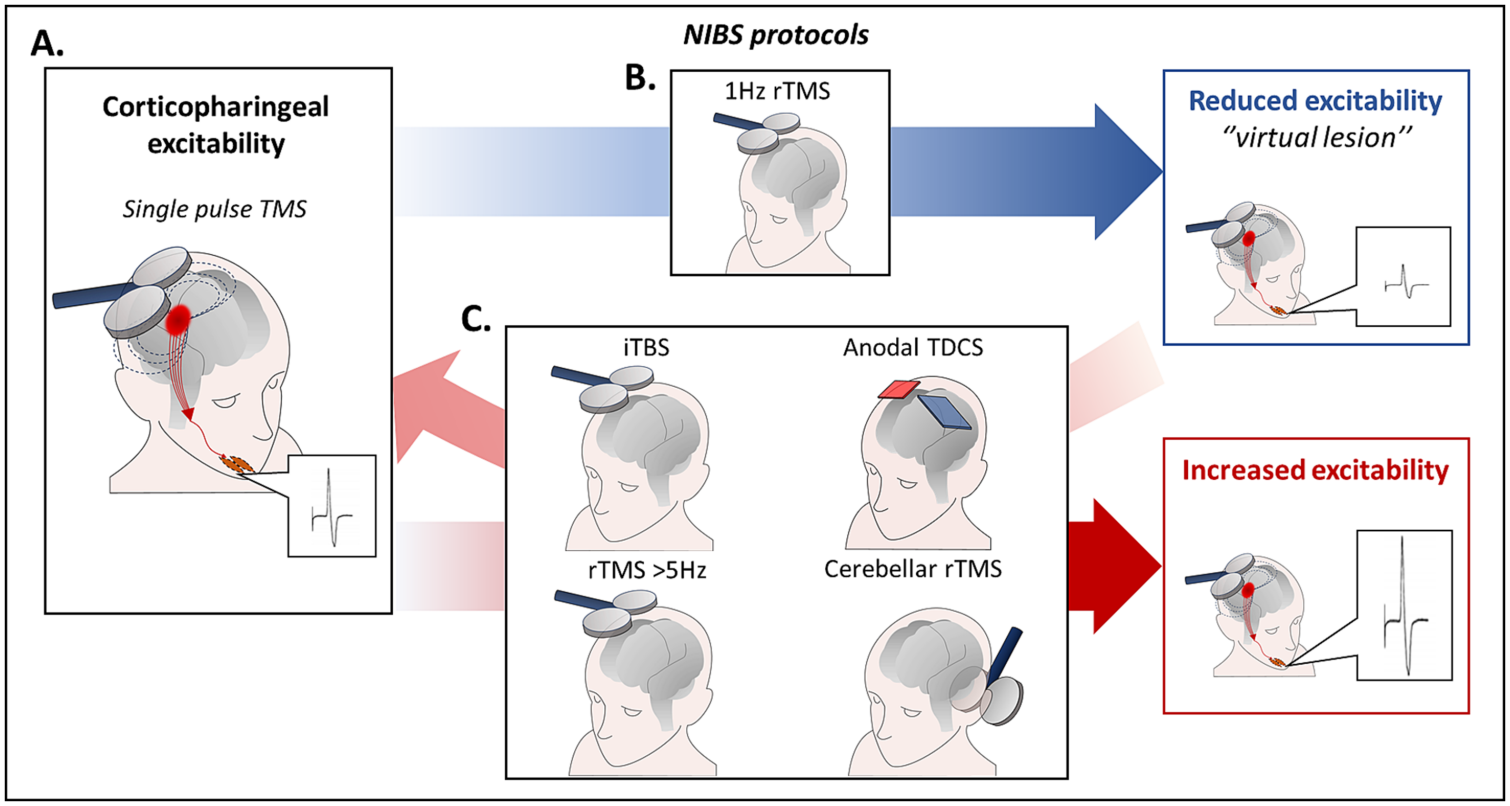
**(A)** Cricopharyngeal excitability can be explored non-invasively using single-pulse TMS. Following stimulation of the pharyngeal (swallowing) motor cortex, MEPs can be recorded from different muscles involved in the oral and pharyngeal phase of swallowing (e.g., submental muscles, cricopharyngeal muscle). **(B)** Low frequency (1 Hz) rTMS of the pharyngeal motor cortex induced a selective temporary suppression of pharyngeal MEPs and altered normal swallowing in healthy subjects, by inducing a “virtual lesion” effect in an experimental setting. **(C)** Different protocols including high frequency (≥ 5 Hz) rTMS, iTBS and anodal TDCS are associated with a long-lasting increase of corticobulbar excitability. In addition, high frequency rTMS can restore cortico-pharyngeal excitability and swallowing impairment induced by 1 Hz rTMS of the hemisphere dominant for swallowing when applied to the contralateral one. Finally, it has been evidenced that also 10 Hz cerebellar rTMS produced an increase of pharyngeal MEPs and was able to revert the effects of a “virtual lesion” of the pharyngeal motor cortex. iTBS, intermittent theta-burst stimulation; MEPs, motor evoked potentials; rTMS, repetitive transcranial magnetic stimulation; TDCS, transcranial direct current stimulation; TMS, transcranial magnetic stimulation.

Single-pulse TMS can be used to explore non-invasively cricopharyngeal excitability. Following stimulation of the pharyngeal (swallowing) motor cortex, motor evoked potentials (MEPs) can be recorded from different muscles involved in the oral and pharyngeal phase of swallowing (e.g., submental muscles, cricopharyngeal muscle) (18–20, 41) (Figure 2A).

Repetitive TMS (rTMS) can be used to produce long-term changes in cortical excitability. rTMS effects depend on the intensity, frequency, and number of stimuli delivered (56, 57). Different rTMS protocols, can be applied to induce transient increases or decreases of cortical excitability resembling mechanisms of LTP and LTD. Low frequency (1 Hz) rTMS of the pharyngeal motor cortex induced a selective temporary suppression of pharyngeal MEPs and altered physiological swallowing in healthy subjects, producing a “virtual lesion” effect (58) (Figure 2B). Conversely, following high frequency (≥ 5 Hz) rTMS a long-lasting increase of cortico-pharyngeal excitability is observed (Figure 2C). Notably, high frequency rTMS was able to restore cortico-pharyngeal excitability and swallowing impairment induced by 1 Hz rTMS of the dominant hemisphere when applied to the contralateral hemisphere (59). Other TMS protocols to induce long lasting increase or decrease of cortical excitability are the intermittent theta-burst stimulation (iTBS) and the continuous TBS (cTBS) (60).

Transcranial direct current stimulation (TDCS) is another widely used technique to induce persistent excitability changes in human brain cortex (61, 62). Whereas the initial effects of stimulation are related to depolarization or hyperpolarization of neuronal membranes (62), the long-lasting changes are due to modulation of NMDA receptors (62, 63). The resulting effects depend on the polarity of stimulation (61), in particular anodal tDCS depolarizes cortical neurons and induces LTP-like effects (62, 63). It has been demonstrated that anodal tDCS of the pharyngeal motor cortex is able to increase cortico-pharyngeal excitability (59), and to enhance sucking activity of liquid bolus in healthy subjects (64).

Different studies have evidenced that non-invasive stimulation of the pharyngeal motor cortex may represent a promising approach for the treatment of stroke patients with dysphagia (65, 66). Systematic meta-analyses of RCTs investigating the effect of NIBS on post-stroke dysphagia showed that rTMS/TBS and tDCS enhanced recovery of swallowing (67–71). Conversely, only few studies have explored the effects of NIBS in MS-related dysphagia (41, 72). It has been reported that anodal tDCS of the swallowing motor cortex produced an improvement of DOSS score in MS patients with mild to moderate dysphagia (72). Moreover, a study in dysphagic MS patients with brainstem involvement, showed that anodal tDCS over swallowing motor cortex for 5 consecutive days was associated with increased cortico-pharyngeal excitability, increased duration of CP muscle relaxation, and a significant reduction of the PAS scores (41).

Finally, as the cerebellum is part of the swallowing motor network, it may represent an important treatment target in MS-related dysphagia. TMS of the cerebellum induces pharyngeal MEPs and potentiates pharyngeal motor output of a subsequent TMS of the swallowing motor cortex (73). In addition, it has been evidenced that high-frequency (10 Hz) rTMS induced an increase of pharyngeal MEPs (74), and was able to revert the effects of the “virtual lesion” of the pharyngeal motor cortex (75). Cerebellar non-invasive stimulation has been increasingly explored for treating dysphagia in patients with stroke (75, 76). However, the role of cerebellar non-invasive stimulation in MS dysphagia has not yet been assessed.

## 5 Discussion

Dysphagia is a very troublesome and life-threatening complication of MS. Early diagnosis and management of swallowing disorders is important to prevent serious outcomes and invasive therapies.

The prevalence of swallowing disorders is higher in older MS patients, and in those with longer disease duration, higher disability, and progressive clinical course (7, 8). However, considering that dysphagia may be present even in the earliest phases of MS and in patients with low disability (2, 6), swallowing difficulties should be early investigated in all patients.

Clinicians should routinely ask about swallowing difficulties and assess for possible indicators of dysphagia, such as weight loss or a history of pneumonia. Specific questionnaires validated for MS-related dysphagia, such as the DYMUS, can help identifying patients with subclinical swallowing impairment or those at risk for developing dysphagia (6, 8). Clinical evaluation and water swallowing tests are also commonly used, although studies in MS are limited (1, 2, 34). Instrumental assessment (i.e., VFS, FEES) represents the gold standard for diagnosing dysphagia, and should performed promptly when dysphagia, even subclinical, is suspected (35, 36).

No specific pharmacological treatments are available for MS dysphagia and management mainly relies on swallowing therapy. Hypo- or hypersalivation could worsen MS dysphagia and may benefit from specific treatment, including botulinum toxin type A (BoNT/A) injection into the major salivary glands (46). Swallowing therapy should be early started, accompanied by regular clinical and instrumental evaluations to quantify the severity of dysphagia using specific clinical scales such as DOSS and PAS. Notably, in patients with severe dysphagia resistant to swallowing therapies, UES hyper-activity may be suspected, warranting consideration of targeted treatments (i.e., surgical myotomy or BoNT/A injection) (47–49).

Both PES and NIBS (rTMS, TBS, tDCS) may represent promising therapeutic approaches for neurogenic dysphagia. Evidence supporting the use of these techniques comes primarily from studies in post-stroke dysphagia, while only few studies have addressed the effects on swallowing disorders in MS. It has been reported that both PES and anodal tDCS of the pharyngeal motor cortex improve swallowing function in dysphagic MS patients (41, 53, 72). However, the evidence supporting the use of these techniques in MS dysphagia is still preliminary.

In conclusion, the early diagnosis of swallowing disorders in MS requires vigilant monitoring by clinicians, including the regular use of questionnaires to periodically screen for these symptoms, as dysphagia can manifest intermittently throughout the course of the disease. Closer monitoring is especially important for high-risk patients, (including older individuals, those with longer disease duration, greater disability, and brainstem, cerebellar, or cognitive symptoms), and should involve early referral for clinical and instrumental evaluations. The treatment of dysphagia in MS remains a problematic issue and alternatives to rehabilitation are limited to the management of complications and associated symptoms. Novel approaches such as PES and NIBS, may play a therapeutic and preventive role and contribute to delay invasive interventions. However, further studies, particularly adequately powered RCTs, are needed to define the contribution of these approaches in MS.

## Data Availability

The original contributions presented in the study are included in the article, further inquiries can be directed to the corresponding author.
