# Corrigendum: Dysphagia in multiple sclerosis: pathophysiology, assessment, and management—an overview

**DOI:** 10.3389/fneur.2025.1613012

**Published:** 2025-05-08

**Authors:** Domenico A. Restivo, Angelo Quartarone, Antongiulio Bruschetta, Angelo Alito, Demetrio Milardi, Rosario Marchese-Ragona, Ennio Iezzi, Sheila Peter, Diego Centonze, Mario Stampanoni Bassi

**Affiliations:** ^1^Department of Clinical and Experimental Medicine, Physical Medicine and Rehabilitation Unit, University of Messina, Messina, Italy; ^2^IRCCS Centro Neurolesi Bonino-Pulejo, Messina, Italy; ^3^Orthopedic Institute of Southern Italy “Franco Scalambrino”, Messina, Italy; ^4^Department of Biomedical and Dental Sciences and Morphofunctional Images, University of Messina, Messina, Italy; ^5^Brain Mapping Lab, Department of Biomedical and Dental Sciences and Morphofunctional Imaging, University of Messina, Messina, Italy; ^6^ENT Department, University of Padua, Padova, Italy; ^7^Unit of Neurology, IRCCS Neuromed, Pozzilli, Italy; ^8^Department of Systems Medicine, University of Rome Tor Vergata, Rome, Italy

**Keywords:** multiple sclerosis, dysphagia, swallowing rehabilitation, non-invasive brain stimulation, pharyngeal electrical stimulation

In the published article, there was an error in **affiliation 2**. Instead of “IRCCS “Fondazione Bonino-Pulejo”, Messina, Italy,” it should be “IRCCS Centro Neurolesi Bonino-Pulejo, Messina, Italy.”

The authors apologize for this error and state that this does not change the scientific conclusions of the article in any way. The original article has been updated.

